# Good Life with osteoArthritis in Denmark (GLA:D™): evidence-based education and supervised neuromuscular exercise delivered by certified physiotherapists nationwide

**DOI:** 10.1186/s12891-017-1439-y

**Published:** 2017-02-07

**Authors:** Søren T. Skou, Ewa M. Roos

**Affiliations:** 1 Research Unit for Musculoskeletal Function and Physiotherapy, Department of Sports Science and Clinical Biomechanics, University of Southern Denmark, 55 Campusvej, DK-5230 Odense M, Denmark; 2 Department of Physiotherapy and Occupational Therapy, Næstved-Slagelse-Ringsted Hospitals, 4200 Slagelse, Region Zealand Denmark

**Keywords:** Osteoarthritis, Exercise therapy, Implementation, Health education

## Abstract

**Background:**

The uptake of evidence-based guidelines in clinical practice is suboptimal in osteoarthritis (OA) and other chronic diseases. Good Life with osteoArthritis in Denmark (GLA:D) was launched in 2013 with the aim of implementing guidelines for the treatment of knee and hip OA in clinical care nationwide. The purpose of this report was to evaluate the effects of the GLA:D intervention from 2013 to 2015, using data from the national GLA:D registry.

**Methods:**

Patients undergo education and supervised exercise delivered by trained physiotherapists. Outcomes evaluated at baseline, 3 and 12 months are pain intensity (0 to 100, best to worst), objective physical function (30-s chair-stand test and 40-m fast-paced walk test), physical activity (number of days per week being physically active for at least 30 min), quality of life (Knee injury and Osteoarthritis Outcome Score (KOOS) and the Hip disability and Osteoarthritis Outcome Score (HOOS) quality of life subscale, 0–100, worst to best), number of patients on painkillers and sick leave, and access to care according to guidelines.

**Results:**

Data from 9,825 participants from the GLA:D registry were utilised in the analyses. It was demonstrated that GLA:D improved pain intensity and quality of life by 12.4 points and 5.4 points at 3 months, and 13.7 points and 9.4 points at 12 months, respectively. Furthermore, physical function and physical activity improved (only at 3 months), fewer patients took painkillers following the treatment, and fewer patients were on sick leave at 12 months following GLA:D compared with the year prior to GLA:D. GLA:D is offered in all five health care regions in Denmark via 286 active GLA:D units, but the uptake in the Danish municipalities is still low with only 20% of the municipalities offering GLA:D.

**Conclusion:**

Three years after its inception, GLA:D has been rolled out nationwide and has a significant impact not only on patient symptoms and physical function, but also on intake of painkillers and sick leave. The lifestyle changes introduced by education and supervised exercise were largely maintained at 1 year and may have the potential to also improve general health and reduce societal costs.

## Background

Chronic diseases such as osteoarthritis (OA), type 2 diabetes, chronic obstructive pulmonary disease and low back pain are highly prevalent and among the leading causes of global disability [1]. These chronic diseases often occur concurrently, making care more complex [2] and potentially leading to worse disability for the individual [3]. An under-appreciated cause of several chronic diseases is physical inactivity [4]. As such, physical activity and exercise represent important components of primary prevention of at least 35 chronic diseases [4]. Furthermore, substantial evidence supports exercise as first line treatment for a range of chronic diseases, including musculoskeletal disorders [5, 6]. However, despite the evidence, physical activity and exercise are under-prescribed in the treatment of chronic diseases, resulting in suboptimal care [7–9].

This is also true for OA. Despite strong evidence from more than 50 randomised, controlled trials (RCTs) in knee OA [10] and 10 RCTs in hip OA [11] supporting the efficacy of exercise, and international guidelines recommending it [12, 13], the uptake of exercise, education and weight loss as first line treatment for patients with painful knee and hip OA is suboptimal in clinical practice [9]. A recent systematic review demonstrated that only 36% of patients with OA received appropriate non-pharmacological care according to the guidelines [9]. Research highlights that the successful implementation of evidence in clinical practice requires a comprehensive approach adapted to the specific setting and stakeholders and designed to address barriers to implementation [14].

Good Life with osteoArthritis in Denmark (GLA:D) was initiated in January 2013 with the overarching aim of implementing clinical guidelines for the treatment of knee and hip OA in clinical practice in order to facilitate high quality care of patients with OA in the Danish population [15]. GLA:D consists of three mandatory elements: a 2-day course for physiotherapists; 8 weeks of education and supervised neuromuscular exercise for patients with knee and hip OA symptoms delivered by a trained physiotherapist in clinical practice; and the national GLA:D registry with data from baseline, 3 and 12 months. The feasibility and short- and long-term improvements in symptoms from GLA:D has previously been demonstrated in a pilot study [16, 17]; however, an evaluation of the effects from GLA:D from a nationwide perspective is lacking.

The purpose of this paper was to evaluate the specific aims of the GLA:D initiative in patients with knee and hip OA. The specific aims are reduced pain, reduced number of patients taking pain killers, improved physical function and physical activity, improved quality of life, and reduced number of patients on sick leave. We also wanted to evaluate the equality of access to care, according to the guidelines across health care sectors and geographic regions in Denmark.

## Methods

### Design

This was a registry-based study evaluating the results from the GLA:D initiative.

Ethics approval was not needed, according to the local ethics committee of the North Denmark Region and neither was clinical trial registration, as it was a register-based study and not a clinical trial. The GLA:D registry was approved by the Danish Data Protection Agency and all patients consented to submitting data to the GLA:D registry.

### Good Life with osteoArthritis in Denmark (GLA:D™)

GLA:D is a non-profit initiative hosted at the University of Southern Denmark. The trademark has been registered to ensure that the quality of care is maintained at a high level, since physiotherapists educated in GLA:D are obliged to follow the principles of GLA:D and contribute data to the national GLA:D registry.

### Course for physiotherapists

Physiotherapists interested in GLA:D participate in a 2-day course giving them the requisite skills to diagnose OA and deliver OA care as described in the clinical guidelines. The course comprises existing evidence on OA and treatment of OA and a thorough introduction to GLA:D, as well as practical instructions in the specific protocol of GLA:D, including delivering patient education, supervising and instructing neuromuscular exercise and the use of the GLA:D registry. On successful completion of the course, the physiotherapist has access to a digital ‘tool box’ with everything needed to start GLA:D at his/her clinic, municipality, or hospital, including access to an online platform with manuals, Power Point presentations for use in patient education, and other relevant material for startup. This ensures that the trained physiotherapists will be able to deliver similar care in accordance with clinical guidelines across the country. Additional treatments are permitted, if the physiotherapist finds them relevant for the individual patient. Information on if and what additional treatments were given is however not recorded in the registry.

### Education and exercise for patients

**Fig. 1:**
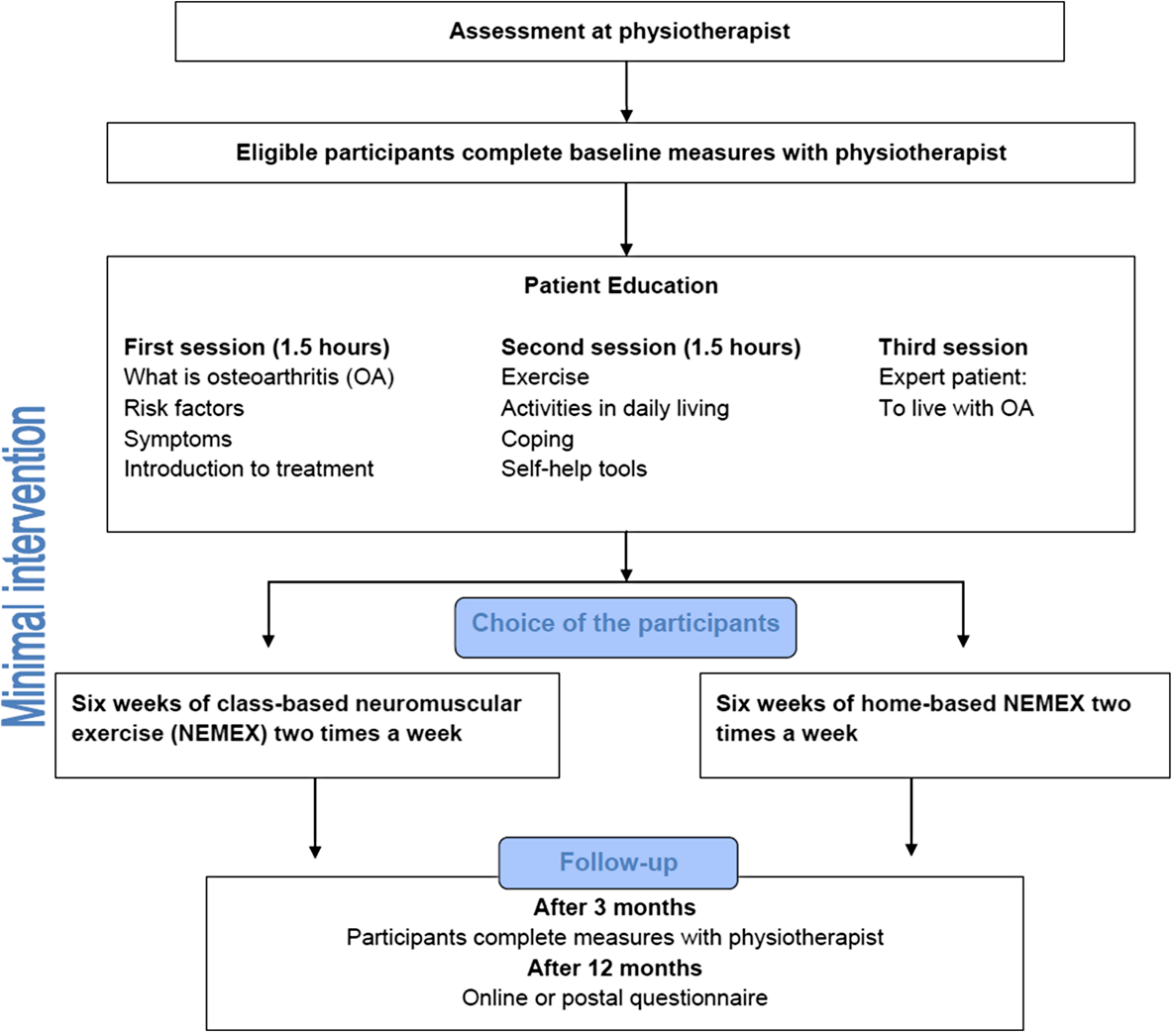
Flow diagram in Good Life with osteoArthritis in Denmark (GLA:D™). Minimal intervention involving education and supervised exercise are mandatory elements of the GLA:D treatment package

GLA:D for patients comprises a ‘minimal intervention’ with three sessions of patient education delivered over 2 weeks and 12 sessions of supervised neuromuscular exercise delivered twice weekly for 6 weeks (Fig. 1). The patient education consists of two sessions given by a trained physiotherapist and one session led by a previous participant in GLA:D (‘expert patient’). The sessions provided by the physiotherapist focus on giving the patient knowledge of OA and treatment of OA with a particular focus on exercise, its beneficial effects on symptoms and general health, and self-help advice. The third session is intended to give the patients the possibility to identify themselves with an expert patient, who has achieved significant improvements in his/her symptoms following GLA:D. All three sessions focus on engaging the patients actively and sharing experiences with each other. Moreover, the patients are strongly encouraged to participate in the group-based NEuroMuscular Exercise programme (NEMEX) [18–22] with 12 sessions each lasting for 60 min. The GLA:D-trained physiotherapists supervise the groups, typically comprising 6–12 patients. Patients who for some reason do not wish to, or are not able to, participate in the supervised exercise can do the exercise programme at home based on detailed instructions by their physiotherapist or combine supervised and home-based exercise. After the 8-week programme, the patient is encouraged to continue being physically active and to exercise, either with their physiotherapist or in their local community, to sustain the effects from the treatment in the long term. Individual strategies for the continuation of physical activity and exercise are discussed at the 3-month follow-up.

### The national GLA:D registry

Data from baseline, 3 and 12 months are registered in the national, electronic GLA:D registry. Data are objectively measured, therapist-reported and patient-reported. The registry is designed to describe the population at baseline, and, after the programme of education and exercise, to evaluate the outcomes of pain, function, quality of life and other outcomes at 3 and 12 months follow up. The outcomes assessed are listed in Tables 1 and 2. The registry contains a core set of outcomes that have been part of the registry since its initiation. Outcomes that are relevant for the patient population (based on emerging evidence) are added, and questions that are no longer relevant are removed. The registry is continuously improved by user involvement meaning that the physiotherapists and patients have influence on database structure, content and questions. Furthermore, data can be made available for the individual therapists and patients with the potential of benchmarking their own results and as motivation for further improvement. A report, outlining the descriptive results, is made publicly available annually, starting in 2013 [15]. Additionally, it is possible to integrate data from the registry with data from the Danish Knee Arthroplasty Registry and the Danish Hip Arthroplasty Registry, among other registries, which offers a unique opportunity to evaluate and improve clinical pathways in this patient population.

**Table 1:** Patient-reported outcomes in Good Life with osteoArthritis in Denmark (GLA:D™) *

Variable	Baseline	3-month follow up	12-month follow up
Gender	X		
Age	X		
Born in Denmark	X		
Danish citizen	X		
Comorbidities	X		
Educational level	X		
Previous injury	X		
Smoking	X		
Live alone/live together with a partner, family, friends or others	X		
Most affected knee/hip joint	X	X	X
Other affected knee/hip joints	X	X	X
Hand/finger problems	X	X	X
Mean pain intensity during the last month in most affected joint	X	X	X
Frequency of knee/hip pain (KOOS/HOOS P1)	X	X	X
Pain mannequin (patients mark areas of the body where they have had pain in the last 24 h)	X	X	X
Walking problems due to knee/hip	X	X	X
Walking problems due to other reasons	X	X	X
Days a week being physically active for at least 30 min	X	X	X
Frequency and duration of exercise	X	X	X
Compared with others of same age, personal level of physical activity	X	X	X
UCLA Activity Score	X	X	X
Fear of movement	X	X	X
Use of pain killers due to knee/hip	X	X	X
Current employment	X	X	X
Sick leave	X	X	X
Home care	X	X	X
SF-12	X	X	X
EQ-5D	X	X	X
KOOS/HOOS QOL including knee/hip confidence	X	X	X
Arthritis Self-Efficacy Scale (subscales: pain and other symptoms)	X	X	X
Knee/hip arthroplasty	X	X	X
Desire for surgery of own knee/hip	X	X	X
Satisfaction with GLA:D		X	X
Frequency of using what was learnt in GLA:D		X	X

^*^ *KOOS* Knee injury and Osteoarthritis Outcome Score, *HOOS* Hip disability and Osteoarthritis Outcome Score, *P1* Item 1 from the subscale Pain from KOOS and HOOS, *UCLA Activity Score* University of California, Los Angeles (UCLA) activity score, *EQ-5D* EuroQol-5 dimension 5 level questionnaire, *QOL* The subscale quality of life from KOOS and HOOS

**Table 2:** Physiotherapist-reported and performance-based outcomes in Good Life with osteoArthritis in Denmark (GLA:D™)

Variable	Baseline	3 months follow-up
Physiotherapist-reported outcomes		
Duration of symptoms	X	
Prior explanation of knee/hip problems	X	
Prior treatment of knee/hip problems	X	
Body Mass Index	X	X
Most affected knee/hip joint	X	X
Other affected knee and hip joints	X	X
Knee and hip surgery	X	X
X-ray of most affected joint during follow-up and signs of osteoarthritis on x-ray	X	X
Using walking aids	X	X
On waiting list for surgery	X	X
Use of painkillers and type	X	X
Participation in patient education sessions and number of supervised exercise sessions in GLA:D	X	X
Other treatment than GLA:D during follow-up	X	X
Performance-based outcomes		
40-m fast paced walk test	X	X
30-s chair-stand test	X	X

### Patients

In Denmark, patients with OA are typically referred to the physiotherapist by their general practitioner in which case approx. 40% of the treatment cost is reimbursed. Patients can also refer themselves directly to the physiotherapist but then have to pay the full treatment cost. Finally, patients can be referred by an orthopaedic surgeon in which case the full treatment cost is reimbursed. Participants may have entered the GLA:D program by all three means. The treating physiotherapist evaluated the inclusion and exclusion criteria. The inclusion criteria for GLA:D are ‘Joint problems from knee and/or hip that have resulted in contact with the health care system’.

The exclusion criteria are: another reason than OA for the problems, for example, tumour; inflammatory joint disease, or sequelae after hip fracture; other symptoms that are more pronounced than the OA problems, for example, chronic, generalised pain, or fibromyalgia.

Radiographs are not needed to diagnose OA according to international [23] and Danish [24] guidelines, and therefore not part of the eligibility criteria for GLA:D.

### Variables

#### Pain intensity

Mean pain intensity during the last month in the most affected joint was evaluated at baseline, and after 3 and 12 months on a 100 mm visual analogue scale (VAS) with terminal descriptors of ‘no pain’ (0 mm) and ‘maximum pain’ (100 mm). The VAS is a measure of pain widely used in patients with OA that is valid, reliable and responsive [25].

#### Intake of painkillers

Intake of painkillers was evaluated by the physiotherapist asking the patients whether they had taken any joint-related medication during the last 3 months, at baseline and after 3 months. If the patients were taking painkillers, they were asked which type and whether or not they were taken because of their knee/hip pain (dichotomised into ‘yes’ or ‘no’ to taking acetaminophen, nonsteroidal anti-inflammatory drugs (NSAIDs) or opioids/opioid-like painkillers).

#### Physical function

Physical function was recorded by the physiotherapists and evaluated using the 30-s chair-stand test and the 40-m fast-paced walk test, which are two tests recommended by the Osteoarthritis Research Society International (OARSI) as components of the minimal core set of performance-based physical function tests for knee and hip OA [26].

#### Physical activity

Physical activity was assessed using a question that the patient answered at baseline, 3 and 12 months about how many days per week that they were physically active for at least 30 min. The results were divided into three groups: physically inactive (0–1 days per week); physically active but not meeting guideline recommendations (2–4 days per week); and physically active and meeting guideline recommendations (5–7 days per week). The 2008 Physical Activity Guidelines Advisory Committee Report recommends at least 2.5 h of physical activity per week, because a lower risk of all-cause mortality and a lower risk of a range of chronic diseases have been consistently observed with this level of physical activity [27].

#### Joint-related quality of life

Joint-related quality of life was evaluated using the subscale quality of life (QOL) from the self-report questionnaires: the Knee injury and Osteoarthritis Outcome Score (KOOS) and the Hip disability and Osteoarthritis Outcome Score (HOOS), with scores ranging from 0 (worst) to 100 (best). KOOS and HOOS are valid, reliable and responsive patient-reported outcome measures previously applied in studies of OA [28–31].

#### Health care visits and costs for the individual patient and society

When a sufficient number of patients had been to the 12-month follow up, a health economic evaluation was conducted. To provide preliminary insight into the effects of GLA:D on the costs related to OA, the number of patients on sick leave were used. Sick leave was evaluated at baseline, 3 and 12 months by asking the patient whether or not they had been on sick leave during the last year due to their knee or hip joint pain, answered with a ‘yes’ or a ‘no’.

#### Access to care across health care sectors and geographic regions

This was evaluated by 1) reporting the number of GLA:D units and 2) relating the number of GLA:D units in the five Regions of Denmark, which had registered patients in GLA:D indicating active GLA:D units, to the self-reported number of patients with OA from the National Health Profile 2013.

### Statistical analyses

All patients with data from the 3-month or 12-month follow ups or a baseline assessment date less than 4 or 13 months before the follow ups were included in the analyses. A one-month delay of the 3-month and the 12-month follow ups was allowed for pragmatic reasons, based on an expected delay for some patients due to unforeseen circumstances.

The analyses of pain intensity, KOOS/HOOS QOL, 30-s chair-stand test and the 40-m fast-paced walk test were conducted using a mixed-effects model (including all available data points) with the patient as a random effect and time (baseline, 3 months; or baseline and 12 months) and joint (knee, hip) as fixed effects. Interaction between time and joint was also included in the model. The analysis was conducted, both unadjusted and adjusted (baseline scores, gender, age, and Body Mass Index (BMI)). A sensitivity analysis was conducted excluding those reporting to have undergone a total joint replacement during follow-up. The results are presented as estimated marginal means with 95% confidence intervals (CI). Furthermore, the results for pain intensity and KOOS/HOOS QOL at baseline, 3 and 12 months are graphically presented.

Ordinal logistic regression analyses (proportional odds models) were applied to investigate whether physical activity level changed from baseline to 3 months and from baseline to 12 months. A sensitivity analysis was conducted excluding those reporting to have undergone a total joint replacement during follow-up. The results are presented as odds ratios (OR) with 95% confidence intervals.

The McNemar’s test was used to assess differences in the number of patients taking painkillers (acetaminophen, NSAIDs or opioids/opioid-like painkillers) at baseline and at 3 months and in the number of patients being on sick leave at baseline and after 12 months. A sensitivity analysis was conducted excluding those reporting to have undergone a total joint replacement during follow-up. Furthermore, the risk differences were calculated. The analyses were conducted for patients with knee and hip OA separately, as differences between the two groups were anticipated. For the analyses of sick leave, only patients associated with the labour market (excluding old-age pensioners and people on early retirement pension or disability pension) were included. Due to small numbers, the analysis was conducted with patients with knee and hip OA combined.

The analyses of differences between patients discontinuing and patients not discontinuing GLA:D were done using unpaired *t*-test and Pearson’s Chi-Square test.

The significance level was set at *P* < 0.05, and all analyses were performed in IBM SPSS Statistics (Version 22, IBM Corporation, Armonk, NY, USA).

## Results

Baseline characteristics of the 9,825 participants with knee and hip OA included in GLA:D between January 31, 2013 and December 31, 2015 are presented in Table 3.

**Table 3:** Baseline characteristics in Good Life with osteoArthritis in Denmark (GLA:D™)^a^

Variable	Knee (*n* = 7 333)	Hip (*n* = 2 492)
Women, *n* (%)	5 405 (74)	1 840 (74)
Age, mean (SD; range) in years	64.0 (9.9; 18–94)	65.5 (9.7; 15–91)
Body mass index, mean (SD; range)	28.4 (5.2; 15–54)	26.7 (4.6; 14–48)
Highest education level completed: Short-term higher education or lower, n (%)	3 206 (48.5)	1 113 (48.3)
Pain intensity during the last month on a 0–100 mm visual analogue scale, mean (SD)	48.2 (22.0)	47.1 (21.8)
Duration of pain, mean (SD) in months	54.7 (79.8)	40.5 (54.4)
Patients using pain medication (acetaminophen, NSAIDs or opioids/opioid-like painkillers) due to their joint pain during the last 3 months, *n* (%)	4 086 (56)	1 478 (59)
30-s chair-stand test, mean number of chair-stands (SD) during 30 s	12.1 (3.7)	12.5 (3.9)
40-m fast-paced walk test, mean (SD) time to complete 40 m in seconds.	28.7 (8.8)	28.7 (8.9)
Patients self-reporting being physically active for at least 30 min at least 5 days a week, *n* (%)	3 976 (60)	1 443 (63)
Joint-related quality of life, from the KOOS/HOOS quality of life subscale, 0–100, worst to best, mean (SD)	44.9 (14.5)	47.3 (14.9)
Patients on sick leave during the last 12 months because of their joint pain, *n* (%)	686 (27)	132 (18)

^a^ *KOOS* Knee injury and Osteoarthritis Outcome Score, *HOOS* Hip disability and Osteoarthritis Outcome Score; the analyses of educational level included 6 613 patients with knee OA and 2 303 patients with hip OA. The analyses of patients on sick leave included only patients with knee (*n* = 2,531) and hip OA (*n* = 736) associated with the labour market (excluding old-age pensioners and people on early retirement pension or disability pension)

### Completeness of GLA:D registry

Nine hundred and thirty-one patients (9%) who had data registered at baseline decided to discontinue the GLA:D programme within the 12 month period, some before ever starting the programme, others during the programme and finally some between the 3 and 12 months follow-ups. The most common reasons for discontinuation were: did not wish to participate after all (23%), could not attend or manage to participate in the treatment (16%), decided to seek other treatment (15%), and could not participate due to own or family illness (14%). The average age (SD) of the patients who discontinued was 64.9 (11.1) and 73% were women. Seventy four percent reported the knee as their primary problem and 52% of the patients who discontinued reported no more than short-term higher education as their highest level of completed education. Average pain (SD) prior to GLA:D for those who discontinued was 52.8 (22.8) for patients with hip OA and 51.6 (23.0) for patients with knee OA, while average BMI was 27.3 (4.4) for patients with hip OA and 28.9 (5.7) for patients with knee OA. While there were no significant difference between patients discontinuing and patients not discontinuing GLA:D in age, proportion of women and proportion reporting the knee as their primary problem (*P* = 0.13–0.89), patients discontinuing had lower levels of completed education, more pain and higher BMI (*P* = 0.001–0.04).

Thus, 8,894 (91%) underwent the GLA:D programme and were eligible for follow up. Out of those eligible for follow up, 84% attended the 3-month follow up and 68% the 12-month follow up. Data from baseline, 3-month and 12-month follow ups were available for 65%.

### Treatment-related variables

Ninety per cent of the patients participated in the first patient education sessions, and 87% in the second. Only 18% participated in the third patient education session run by an expert patient, primarily owing to the fact that several new GLA:D units still had not found an expert patient to run this session as they had only recently finished their first group of GLA:D patients. Eighty-three per cent of the patients with knee OA and 84% of the patients with hip OA participated in at least 10 supervised exercise sessions, while only 4% of the patients with knee OA and 3% of the patients with hip OA chose not to participate in the supervised group-based exercise at all. During the 12-month follow up, 15% of the patients with hip OA and 5% of the patients with knee OA had undergone a total joint replacement of their most affected joint, while 3% of the patients with hip OA and 2% of the patients with knee OA had undergone a total joint replacement of another hip or knee joint.

### Pain intensity

From baseline to 3 months, the patients (*n* = 7,247) reported a crude mean improvement (95% CI) of 12.4 mm (11.8 to 13.1) and a similar adjusted (*n* = 7,189) mean improvement (95% CI) of 12.4 mm (11.8 to 13.1) in pain intensity. Including only data from patients who had not undergone a total joint replacement (*n* = 7,116), the adjusted mean improvement (95% CI) was 12.3 mm (11.7 to 13.0). In all three analyses, a significant interaction was demonstrated between follow-up time and joint (*P* < 0.001) with larger improvements from baseline to 3 months in patients with knee OA compared with patients with hip OA. In the adjusted analysis of all patients, the patients with knee OA improved (95% CI) from 48.1 mm (47.5 to 48.7) to 34.3 mm (33.6 to 34.9), while the patients with hip OA improved (95% CI) from 47.1 mm (46.1 to 48.1) to 36.1 mm (35.0 to 37.1).

From baseline to 12 months, the patients (*n* = 3,431) reported a crude mean improvement (95% CI) of 13.7 mm (12.6 to 14.9) and a similar adjusted (*n* = 3,402) mean improvement (95% CI) of 13.7 mm (12.6 to 14.9) in pain intensity. Including only data from patients who had not undergone a total joint replacement (*n* = 3,210), the adjusted mean improvement (95% CI) was 12.0 mm (10.8 to 13.2).

Only in the analysis without patients who had undergone a total joint replacement was a significant interaction demonstrated between follow-up time and joint (*P* = 0.03) with larger improvements from baseline to 12 months in patients with knee OA compared with patients with hip OA. In this analysis, the patients with knee OA improved (95% CI) from 47.3 mm (46.4 to 48.1) to 33.9 mm (32.7 to 35.0) while the patients with hip OA improved (95% CI) from 46.3 mm (44.8 to 47.8) to 35.6 mm (33.7 to 37.6).

**Fig. 2:**
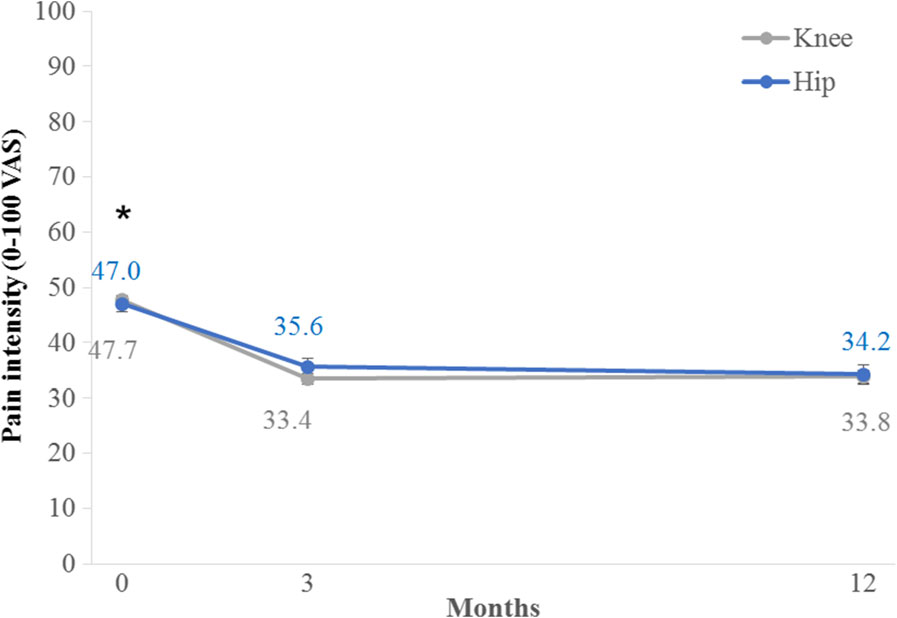
Pain intensity at baseline, 3 and 12 months in patients with knee and hip osteoarthritis participating in Good Life with osteoArthritis in Denmark (GLA:D™). Adjusted (baseline pain intensity, gender, age, and BMI) estimated marginal means from a mixed effects model (*n* = 3,402). Error bars indicate 95% confidence intervals. Pain intensity was significantly lower at 3 and 12 months compared with baseline (*; *P* < 0.001). VAS = Visual Analogue Scale

Figure 2 illustrates the improvements in pain intensity from baseline to 3 and 12 months when including all patients in an adjusted analysis.

### Intake of painkillers

#### Patients with knee OA

From baseline to 3 months (*n* = 4,023), significantly fewer patients with knee OA took acetaminophen, NSAIDs or opioids/opioid-like painkillers (*P* < 0.0001) with a risk of taking painkillers (95% CI) of 55.9% (54.4–57.5) at baseline and 36.7% (35.2–28.2) at 3 months, corresponding to a risk reduction of 19.2%. This remained significant (*P* < 0.0001; *n* = 3 994) when excluding those who had undergone a total knee replacement with a similar risk of taking painkillers (95% CI) of 55.9% (54.4–57.5) at baseline and 41.5% (40.0–43.1) at 3 months, corresponding to a risk reduction of 19.3% (Table 4).

**Table 4:** Patients taking painkillers and being on sick leave at baseline and follow up^a^

Baseline to 3 months				
Outcome	Joint	Baseline status	Yes at 3 months	No at 3 months
Painkillers due to knee/hip?	Knee (*n* = 4,023)	Yes at baseline (*n* = 2,250)	1,162	1,088
		No at baseline (*n* = 1,773)	315	1,458
		Risk of taking painkillers at baseline (95% CI)	55.9% (54.4–57.5)	
		Risk of taking painkillers at 3 months (95% CI)	36.7% (35.2–28.2)	
	Hip (*n* = 1,385)	Yes at baseline (*n* = 804)	472	332
		No at baseline (*n* = 581)	147	434
		Risk of taking painkillers at baseline (95% CI)	58.1% (55.5–60.7)	
		Risk of taking painkillers at 3 months (95% CI)	44.7% (42.1–47.3)	
Baseline to 12 months				
Outcome	Joint	Baseline status	Yes at 12 months	No at 12 months
Sick leave due to knee/hip?*	Knee and hip (*n* = 711)	Yes at baseline (*n* = 173)	53	120
		No at baseline (*n* = 538)	53	485
		Risk of being at sick leave at baseline (95% CI)	24.3% (21.2–27.5)	
		Risk of being at sick leave at 12 months (95% CI)	14.9% (12.3–17.5)	

^a^ Painkillers were defined as acetaminophen, NSAIDs or opioids/opioid-like painkillers; only patients associated with the labour market (excluding old-age pensioners and people on early retirement pension or disability pension) were included in the analysis of sick leave. The risk of taking pain killers at 3 months and the risk of being on sick leave at 12 months were significantly lower than the corresponding risks at baseline (*P* < 0.0001) for patients with knee and hip OA, respectively

#### Patients with hip OA

From baseline to 3 months (*n* = 1,385), significantly fewer patients with hip OA took acetaminophen, NSAIDs or opioids/opioid-like painkillers (*P* < 0.0001) with a risk of taking painkillers (95% CI) of 58.1% (55.5–60.7) at baseline and 44.7% (42.1–47.3) at 3 months, corresponding to a risk reduction of 13.4%. This remained significant (*P* < 0.0001; *n* = 1,361) when excluding those who had undergone a total knee replacement with a risk of taking painkillers (95% CI) of 57.8% (55.2–60.5) at baseline and 44.5% (41.8–47.1) at 3 months, corresponding to a risk reduction of 13.4% (Table 4).

The number of patients taking/not taking painkillers at baseline and 3 months are presented in Table 4.

### Objectively assessed physical function

#### 30-second chair-stand test

From baseline to 3 months, patients (*n* = 7,537) had a crude mean improvement (95% CI) of 2.3 (2.2 to 2.4) and a similar adjusted (*n* = 7,433) mean improvement (95% CI) of 2.3 (2.2 to 2.4) chair-stands during 30 s. Including only patients who had not undergone a total joint replacement (*n* = 7,363), the adjusted mean improvement (95% CI) was similar at 2.3 (2.2 to 2.4).

#### 40-metre fast-paced walk test

From baseline to 3 months, patients (*n* = 7,478) had a crude mean improvement (95% CI) of 2.5 (2.3 to 2.7) and a similar adjusted (*n* = 7,367) mean improvement (95% CI) of 2.5 (2.3 to 2.7) seconds to complete the 40-m fast-paced walk test. Including only data from patients who had not undergone total joint replacement (*n* = 7,297), the adjusted mean improvement (95% CI) was similar at 2.5 (2.3 to 2.6).

### Patient-reported physical activity

The odds of being more physically active at 3 months compared with that at baseline was 1.18 (1.10 to 1.27), which was significant: *P* < 0.0001 (*n* = 7,273). This remained significant (*P* < 0.0001; *n* = 7,199) when excluding those who had undergone a total joint replacement, with identical odds of being more physically active at 3 months compared with that at baseline of 1.18 (1.10 to 1.27).

The odds of being more physically active at 12 months compared with that at baseline was 1.10 (0.99 to 1.23), which was not significant: *P* = 0.09 (*n* = 3,429). This remained non-significant (*P* = 0.16; *n* = 3,235) when excluding those who had undergone a total joint replacement, with the odds of being more physically active at 12 months compared to that at baseline being 1.08 (0.97 to 1.22).

The percentage of patients being physically inactive (0–1 days per week with at least 30 min of physical activity), physically active but not meeting guideline recommendations (2–4 days per week), and physically active and meeting guideline recommendations (5–7 days per week) at baseline compared with that at 3 and 12 months are presented in Table 5.

**Table 5:** Patient physical activity levels at baseline and follow-up^a^

Baseline to 3 months (*n* = 7,273)		
Physical activity level	Percentage at baseline	Percentage at 3 months
Physically inactive	8.1%	3.8%
Physically active but not meeting guideline recommendations	30.6%	32.4%
Physically active and meeting guideline recommendations	61.3%	63.8%
Odds of being more physically active at 3 months compared to at baseline (95% CI)	1.18 (1.10 to 1.27)	
Baseline to 12 months (*n* = 3,429)		
Outcome	Percentage at baseline	Percentage at 12 months
Physically inactive	8.2%	4.3%
Physically active but not meeting guideline recommendations	29.8%	32.7%
Physically active and meeting guideline recommendations	62.1%	63.0%
Odds of being more physically active at 12 months compared to at baseline (95% CI)	1.10 (0.99 to 1.23)	

^a^ The proportion of patients being physically inactive (0–1 days per week with at least 30 min of physical activity), physically active but not meeting guideline recommendations (2–4 days per week), and physically active and meeting guideline recommendations (5–7 days per week)

### Joint-related quality of life

From baseline to 3 months, patients (*n* = 7,258) reported a crude mean improvement (95% CI) of 5.4 (5.0 to 5.9) and a similar adjusted (*n* = 7,206) mean improvement (95% CI) of 5.4 (5.0 to 5.9) in KOOS/HOOS QOL. Including only data from patients who had not undergone a total joint replacement (*n* = 7,132), the adjusted mean improvement (95% CI) was similar at 5.4 (4.9 to 5.8). In all three analyses, a significant interaction was demonstrated between follow-up time and joint (*P* < 0.001) with larger improvements in patients with knee OA compared with patients with hip OA. In the adjusted analysis of all patients, the patients with knee OA improved (95% CI) from 44.9 (44.5 to 45.3) to 51.1 (50.7 to 51.6) units while the patients with hip OA improved (95% CI) from 47.1 (46.4 to 47.7) to 51.7 (50.9 to 52.5) units on a 0–100 worst to best scale.

From baseline to 12 months, the patients (*n* = 3,434) reported a crude mean improvement (95% CI) of 9.4 (8.6 to 10.2) and a similar adjusted (*n* = 3,401) mean improvement (95% CI) of 9.4 (8.6 to 10.2) units in KOOS/HOOS QOL. Including only data from patients who had not undergone a total joint replacement (*n* = 3,208), the adjusted mean improvement (95% CI) was 8.2 (7.3 to 9.0).

**Fig. 3:**
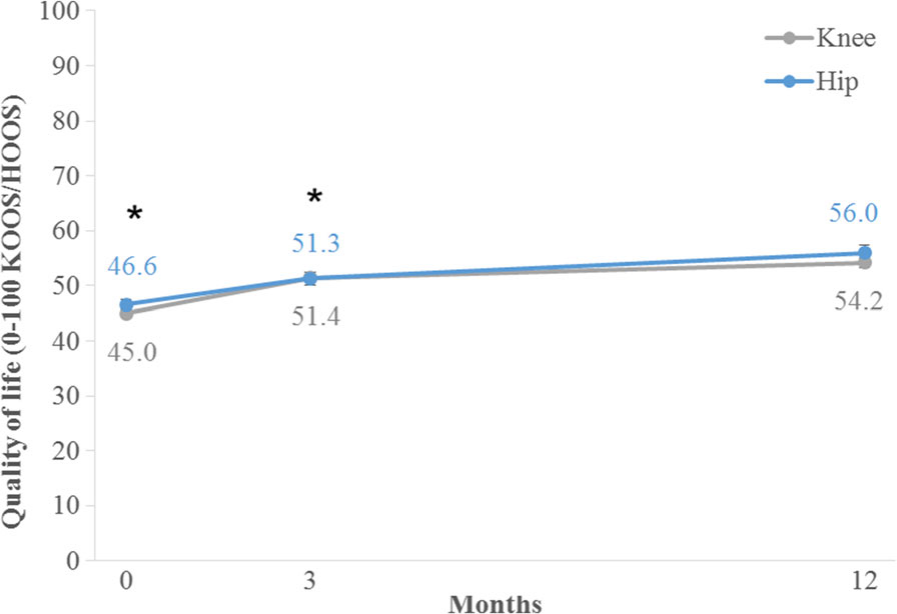
Joint-related quality of life (KOOS/HOOS QOL) at baseline, 3 and 12 months in patients with knee and hip osteoarthritis participating in Good Life with osteoArthritis in Denmark (GLA:D™). Adjusted (baseline KOOS/HOOS QOL, gender, age, and BMI) estimated marginal means from a mixed effects model (*n* = 3,405). Error bars indicate 95% confidence intervals. QOL was significantly higher at 3 and 12 months compared with baseline (*; *P* < 0.001) and at 12 months compared with 3 months (^#^; *P* < 0.001). KOOS = Knee injury and Osteoarthritis Outcome Score; HOOS = Hip disability and Osteoarthritis Outcome Score

Figure 3 illustrates the improvements in KOOS/HOOS QOL from baseline to 3 and 12 months when including all patients in an adjusted analysis.

### Patient-reported sick leave

From baseline to 12 months (*n* = 711), significantly fewer patients were on sick leave (*P* < 0.0001) compared with the year prior to their participation in GLA:D. The risk of being on sick leave (95% CI) was 24.3% (21.2–27.5) at baseline and 14.9% (12.3–17.5) at 12 months, corresponding to a risk reduction of 9.4%. This remained significant (*P* < 0.0001; *n* = 623) when excluding those who had undergone a total joint replacement, with a risk of being on sick leave (95% CI) of 24.3% (21.0–27.6) at baseline and 9.8% (7.5–12.1) at 12 months, corresponding to a risk reduction of 14.5% (Table 4).

The number of patients being on sick leave at baseline compared with that at 12 months are presented in Table 4.

### Access to care across health care sectors and geographic regions

In the second quarter of 2016, there were 4.7 million citizens who were 16 years or older in Denmark [32]. Based on percentages of patients with self-reported OA (not restricted to knee and hip OA) from the National Health Profile 2013 [33], 932,563 citizens of 16 years and above have OA in Denmark.

**Fig. 4:**
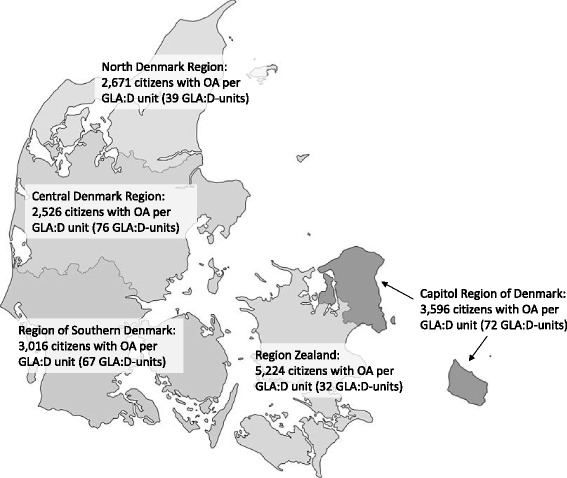
Distribution of clinical units in Good Life with osteoArthritis in Denmark (GLA:D™). The GLA:D units across the five regions of Denmark, which had registered patients in the GLA:D registry, and the number of citizens of 16 years and above with self-reported osteoarthritis (OA) per GLA:D unit. Picture of Denmark has been modified from Jarke [45] licensed under the Creative Commons Attribution-Share Alike 2.5 Generic License

As of May 2016, there were 286 active GLA:D units across Denmark, which had registered patients in GLA:D, suggesting a patient to clinic ratio of 3,229 citizens with self-reported OA per GLA:D unit in Denmark. Figure 4 illustrates the distribution of active GLA:D units across the five regions of Denmark and the number of citizens of 16 years and above with self-reported OA per GLA:D unit. The number of GLA:D units has increased exponentially each year since 2013.

The most active GLA:D units are private physiotherapy clinics (*n* = 257), where patients pay 60-100% of the treatment cost. Only 20 municipalities (with 26 GLA:D units) of the 98 Danish municipalities offering physiotherapy treatment free of charge to the patient (20%) have registered patients in GLA:D (physiotherapists from 32 municipalities have been on the GLA:D course). The last active GLA:D units are two at private hospitals, most often paid by either the government or a private health insurance, and one GLA:D unit based at a public hospital.

## Discussion

Patients with knee and hip OA participating in education and supervised exercise delivered nationwide in clinical practice experience improved pain, physical function, physical activity (only in the short term) and quality of life in the short term and long term as compared to before participating in the treatment programme. Furthermore, fewer patients were taking painkillers after the treatment as compared with before, and fewer patients were on sick leave 12 months after the treatment as compared with before the treatment. Albeit the treatment is offered in all five health care regions of Denmark, the uptake in the Danish municipalities, where the program is free of cost to the participant, is still low. This suggests there is room for improvement to ensure that all patients, including those who potentially do not participate due to a financial barrier, are offered evidence-based care for osteoarthritis.

Exercise is feasible and effective in patients at all severity levels of OA [34], even in those with moderate to severe OA eligible for total knee and total hip replacement [20–22, 35]. Furthermore, if the patient progresses to surgery, supervised exercise prior to surgery seems appropriate since it is associated with a faster post-operative recovery [36]. In two recent systematic reviews and meta-analyses of 54 randomised controlled trials of exercise in patients with knee OA [10] and 10 in patients with hip OA [11], exercise was demonstrated to reduce pain and improve function and quality of life in patients with knee OA, but not in patients with hip OA, immediately after the treatment. The treatment effects were sustained for least 2 to 6 months after the treatment period [10, 11]. Although without a control group for comparison, our study confirms these findings from randomized controlled trials can be transferred into real-life clinical practice. We found that education and exercise delivered through GLA:D improved pain by 13.8 points for patients with knee OA and 11.0 points for patients with hip OA immediately after the treatment, while joint-related quality of life improved by 6.2 points in the patients with knee OA and 4.6 points in the patients with hip OA. Furthermore, the effects translated into objectively measureable positive effects in gait speed and ability to transfer from sitting to standing, while only a small, immediate, self-reported effect on physically activity level was demonstrated. We found that the effects seen immediately after GLA:D were sustained or even further improved at 12 months compared with 3 months. We speculate that the educational sessions are a key component to the sustained results 9 months after the treatment programme ended, as participants learn about the disease, how to self-manage, and the importance of life-long exercise and physical activity for sustained effects. Furthermore, the educational sessions address common misconceptions of exercise being detrimental to the OA joint, and that joints with more severe radiographic OA severity are less likely to benefit from exercise. This speculation is supported by a recent review, highlighting the importance of patient education in order to improve patient adherence to exercise [37].

An interesting finding was the reduced risk of taking painkillers following GLA:D compared with before GLA:D. Our results confirm similar within-group findings from two randomised controlled trials where one treatment arm received a combined treatment programme including the same exercise and educational programme as in GLA:D [21, 22]. Since acetaminophen, NSAIDs and opioids are associated with an increased risk of serious adverse events [12], while adverse events related to exercise are uncommon, mild and temporary, even in patients with moderate to severe OA [18, 38], our findings hold promise, not only due to the pain-relieving effects, but also since it has the potential of reducing serious adverse events in the community.

Another promising finding of our study is the reduced risk of sick leave 1 year after GLA:D compared with before GLA:D. Patients with knee OA have twice the risk of being on sick leave and 40–50% increased risk of receiving a disability pension as compared with the general population, with approximately 2% of all sick days in the society being due to knee OA [39]. It is important to recognize that since we did not include a control group, we cannot attribute the change in sick leave to the GLA:D programme. In theory, societal and other changes may well influence rates of sick leave. However, the findings are encouraging and suggest that offering a treatment programme of education and exercise therapy to all patients with OA may have a huge impact on the societal burden from osteoarthritis.

As illustrated in Fig. 4, each region of Denmark has a large number of GLA:D units. However, the number of potential OA patients per unit varies across the regions spanning from 2,000 citizens to 4,179 citizens. Whether an optimal number of citizens per unit exists is uncertain, but the numbers highlight that the availability of GLA:D is different across the regions. Since the number of GLA:D units has increased rapidly each year, the number of patients per unit is expected to be reduced in the future. However, most of the GLA:D units are private, where patients pay most of the treatment costs themselves, while only one in five Danish municipalities are offering GLA:D at no charge to patients. As lower socio-economic status is associated with an increased risk of OA [40], and since socio-economic disparities exist when it comes to accessing treatments including education and exercise [41], this might also affect the generalizability of our findings. A future focus for the Danish municipalities should be to ensure that all patients have access to GLA:D to overcome potential financial barriers to participation.

Some limitations should be mentioned. First of all, the lack of a control group most probably results in an overestimation of the specific treatment effects when compared to the effects seen in controlled clinical trials, as the contextual effects contributing to the overall treatment effect cannot be determined [42]. The effect perceived by a patient in clinical practice however consists of both the specific and unspecific treatment effects. Secondly, a large proportion (62%) of the patients were already at baseline physically active at least 30 min per day 5 days a week, which is somewhat higher than presented in a systematic review demonstrating that in other countries 41% of patients with knee OA and 58% with hip OA complete at least 150 min. of moderate to vigorous physical activity a week [43]. Danish participants being more physically active already at baseline could be part of the explanation for the small and only short-term improvement in physical activity seen. It should also be noted that we used self-report of physical activity which is known to be difficult and biased [44]. Thirdly, patients discontinuing GLA:D had lower levels of completed education, more pain and higher BMI than patients not discontinuing. Albeit, the differences were relatively small, this suggests that more attention should be given to specific subgroups in GLA:D to support their continuation of the programme. Lastly, the analyses are based on registry-based data collected in clinical practice as opposed to the rigorous, controlled setting of a randomised, controlled trial, thereby reflecting wider variations in treatment protocols and data collection procedures for objectively-measured outcomes. However, the results support implementation of education and exercise in a real-life clinical setting to improve the symptoms of patients with knee and hip OA worldwide.

## Conclusions

Education and supervised exercise delivered nationwide in Denmark in clinical practice through GLA:D appeared to improve pain, physical function, physical activity and quality of life and reduce the number of patients taking painkillers and being on sick leave. As GLA:D includes structured, supervised exercise and advice on physical activity, it has the potential to not only improve OA symptoms, but also to positively affect other chronic diseases and improve the general health and well-being of Danish citizens.

## Abbreviations

BMI: Body mass index
CI: Confidence interval
GLA:D: Good Life with osteoArthritis in Denmark
HOOS: The Hip disability and Osteoarthritis Outcome Score (HOOS)
KOOS: The Knee injury and Osteoarthritis Outcome Score
NEMEX: NEuroMuscular Exercise programme
NSAIDs: Nonsteroidal anti-inflammatory drugs
OA: Osteoarthritis
OARSI: The Osteoarthritis Research Society International
QOL: Quality of life subscale of KOOS/HOOS
RCT: Randomised, Controlled Trial
SD: Standard deviation
VAS: Visual analogue scale
